# Advances in Clinical Decision Support Systems: Contributions from the 2024 Literature

**DOI:** 10.1055/s-0046-1816620

**Published:** 2026-11-03

**Authors:** Lipika Samal, Christoph U. Lehmann

**Affiliations:** 1 Brigham and Women's Hospital, Boston, MassachusettsUSA; 2 Harvard Medical School, Boston, MassachusettsUSA; 3 The University of Texas Southwestern Medical Center, Dallas, TexasUSA

**Keywords:** medical informatics, clinical decision support system, implementation, expert system

## Abstract

**Objective** : To summarize significant research contributions published in 2024 in the field of clinical decision support (CDS) systems and select the best papers for the Decision Support section of the International Medical Informatics Association (IMIA) Yearbook 2025.

**Methods** : A renewed search query for identifying CDS scholarship was developed using Medical Subject Headings (MeSH) terms and related keywords. The query was executed in PubMed in January 2025. The search results were reviewed in three stages by two reviewers: title-based triaging, followed by abstract screening, and then full text review. The resulting articles were sent for external review to identity best paper candidates.

**Results** : A total of 3,092 articles related to CDS were retrieved. Of these, eight articles were selected as candidates for best papers. The general themes of the final three best papers are i) integrating pharmacogenetic data in commercial electronic health record, ii) reducing anesthetic fresh gas flow to improve environmental sustainability, and iii) decreasing activities and interventions overnight in hospitalized patients.

**Conclusion** : Contributions to the field of decision support continued to grow in 2024, therefore making the selection process for the best candidate papers more labor-intensive.

## 1. Introduction

Clinical decision support (CDS) systems are central to promote safe and evidence-based decision in the increasingly complex healthcare ecosystem. This manuscript represents the synopsis for the Decision Support section of the 2025 International Medical Informatics Association (IMIA) Yearbook. The synopsis supplements the review paper on CDS related to climate change, authored by Schleyer *et al.* [ 1 ]. The aim of this synopsis is to summarize significant research in the CDS domain and to select the best papers published in this field in the year 2024.

## 2. Methods

We used a search strategy that we developed for the CDS synopsis in the 2024 IMIA Yearbook and modified the period from the calendar year 2023 to 2024 [ 2 ]. We are including the actual query in Table 1 to support future Yearbook contributors. Like the prior synopsis, we searched for relevant papers in PubMed® using the revised search strategy. We included journal articles published in 2024 in English that had a series of keywords either in the MeSH terms or in the paper's title or abstract. For the MeSH terms, we considered, “Clinical Decision Support”, “Expert systems”, Medical Order Entry Systems”, “Decision Support Techniques”, and “Decision Support Systems, Management”. For the abstracts and title search, we used the terms, “Best Practice Alert”, “Clinical Decision Support”, “Decision Support Techniques”, “Decision Support Systems”, “Expert systems”, “Medication Alert Systems”, and “Computerized Provider Order Entry System”. The final query is shown in Table 1 . We manually excluded journals listed in Beall's List of Potential Predatory Journals and Publishers ( https://beallslist.net/ ).

**Table 1.** Search Strategy for CDS Literature Review

Query
2024[DP] NOT pubstatusaheadofprint AND (journal article[PT]) AND English[LA] AND ( (Clinical Decision Support[MeSH Terms]) OR (Expert systems[Mesh]) OR (Medical Order Entry Systems[Mesh]) OR (Decision Support Techniques[MeSH Terms]) or (Decision Support Systems, Management [MeSH Terms]) OR “Best Practice Alert”[title/abstract] OR “Clinical Decision Support”[title/abstract] OR “Decision Support Techniques”[title/abstract] OR “Decision Support Systems”[title/abstract] OR “Expert systems”[title/abstract] or “Medication Alert Systems”[title/abstract] OR “Computerized Provider Order Entry System” [title/abstract] )

For several years, five categories of CDS were included: i) New methods for CDS, ii) Applications of CDS, iii) Evaluations of a CDS, iv) Reviews on the design of CDS, and v) Systematic reviews/meta-analyses of CDS impact [ 3 ]. In contrast, we maintained the approach of the 2024 Yearbook synopsis where we only included the first three categories.

We reviewed the query results in three stages: title only review, followed by abstract review, and then full text review as described below. We chose to first triage using a title only review because of the volume of results from the query (see Results section for more details). At this stage, we were blinded to country of origin, institution, or authors. We included any articles that were selected for further review by at least one of section editors (CUL and LS). Following the title only review, we proceeded to screen the remaining articles using their abstracts. We then individually examined the full text of all remaining articles and held a virtual discussion of the eleven articles that were selected by both editors. Through consensus, we identified the final eight candidate best papers presented to the full IMIA Yearbook editorial board. The editorial board voted and selected the three best paper finalists.

## 3. Results

The search query resulted in 3,092 unique references. After the title review, 116 references were included by either section editor (CUL and LS). After screening the abstracts, 46 articles were identified with six selected by both editors. After full text review, eight best paper candidates were selected by consensus ( Table 2 ) [ 4 5 6 7 8 9 10 11 ]. After external review of eight best paper candidates, the IMIA Yearbook editorial committee finally selected three candidates as the best papers ( Table 3 ) with the most significant contributions in the field of clinical decision support for 2024. The three finalist papers were published in Frontiers in Pharmacology, the Journal of Graduate Medical Education, and Applied Clinical Informatics.

**Table 2.** Best paper candidates for the Clinical Decision Support Section

PMID	Title	First Author	Journal	DOI
39759455*	Integration of pharmacogenetic data in epic genomic module drives clinical decision support alerts	Newsom KJ	Frontiers in Pharmacology	10.3389/fphar.2024.1458095
39677900*	Implementing a Clinical Decision Support Tool to Reduce Operating Room Anesthetic Fresh Gas Flow: A Resident-Led, Sustainability-Focused Quality Improvement Initiative	Collins J	Journal of Graduate Medical Education	10.4300/jgme-D-24-00074.1
39191425*	Nudging towards Sleep-Friendly Health Care: A Multifaceted Approach on Reducing Unnecessary Overnight Interventions	Kadura S	Applied Clinical Informatics	10.1055/a-2404-2344
38452289	Why do users override alerts? Utilizing large language model to summarize comments and optimize clinical decision support	Liu S	Journal of the American Medical Informatics Association	10.1093/jamia/ocae041
38996876	Implementing a clinical decision support tool to increase early peanut introduction guidance	Rowland AF	Journal of Allergy and Clinical Immunology	10.1016/j.jaci.2024.07.005
38788772	Leveraging Novel Clinical Decision Support to Improve Preferred Language Documentation in a Neonatal Intensive Care Unit	Mercado O	Applied Clinical Informatics	10.1055/a-2332-5843
39411867	Pediatric High Blood Pressure Recognition Associated With Electronic Decision Support: A Cohort Analysis	Zachariah JP	Hypertension	10.1161/hypertensionaha.124.23532
38086417	Addressing Alert Fatigue by Replacing a Burdensome Interruptive Alert with Passive Clinical Decision Support	Fallon A	Applied Clinical Informatics	10.1055/a-2226-8144
*Best paper finalist				

**Table 3.** Selection of best papers for the 2025 IMIA Yearbook of Medical Informatics for the section Decision Support. The articles are listed in alphabetical order of the first author's last name.

Section: Decision Support
• Collins J, Karim M, Akcay B, Palaniappa N, Wong J. Implementing a Clinical Decision Support Tool to Reduce Operating Room Anesthetic Fresh Gas Flow: A Resident-Led, Sustainability-Focused Quality Improvement Initiative. J Grad Med Educ. 2024 Dec;16(6 Suppl):140-144. https://doi.org/10.4300/JGME-D-24-00074.1 . Epub 2024 Dec 13. PMID: 39677900; PMCID: PMC11644587. • Kadura S, Eisner L, Lopa SH, Poulakis A, Mesmer H, Willnow N, Pigeon WR. Nudging towards Sleep-Friendly Health Care: A Multifaceted Approach on Reducing Unnecessary Overnight Interventions. Appl Clin Inform. 2024 Oct;15(5):1025-1039. https://doi.org/10.1055/a-2404-2344 . Epub 2024 Aug 27. PMID: 39191425; PMCID: PMC11602248.

## Discussion and Outlook

The best paper candidates cover a range of clinical conditions and clinical settings. Similarly to the previous year, the majority of the CDS systems were clinician-centered, with limited to no involvement of patients or patient-facing CDS.

The three best paper finalists illustrate innovative approaches to improve quality, patient safety, patient-centeredness, and environmental sustainability. Newsom *et al.* [ 4 ] describe a locally-developed middleware solution to improve the reporting of pharmacogenetic information and associated clinical decision support in a commercial electronic health record (Epic). The project includes automation and adherence to HL7 standards, which broadens the applicability of the approach. The authors included enough detail to make it feasible for another institution to adopt the same approach. The paper provides an example of what could become the standard of care in the future. The other two papers also provide generalizable knowledge and are strengthened by rigorous evaluations of the interventions. Collins *et al.* [ 5 ] describe a quality improvement initiative to reduce the environmental effect of unnecessary anesthetic gas emissions. Clinicians were alerted when fresh gas flow exceeded 1 liter/minute. The intervention reduced sevoflurane usage by 37%. Of note, the project was led by residents (physicians-in-training). Kadura *et al.* [ 6 ] describe the success of a project with the goal of reducing overnight medication administrations and vitals checks. The team addressed multiple aspects of orders in the intervention including nudges through EHR modifications by altering defaults, introducing visible sleep-friendly options, and pharmacists providing Secure Chat reminders. The evaluation included consideration of unintended consequences: the team monitored adverse events in the post-implementation period.

Though the literature review did not identify many examples of clinician-facing CDS for environmental sustainability, it is remarkable that one of them was selected as a best paper finalist. Our prediction for the next year is that there will be more examples of artificial intelligence and specifically large language model CDS. We hope that there will be representation across category I, New methods for CDS, and category II, Applications of CDS, but also category III, Evaluations of CDS. We and other Yearbook editors have put forth a paper outlining 16 key principles for artificial intelligence, encompassing ethical, technical, and organizational domains [ 12 ]. The authors include consideration of the AI lifecycle and draw attention to the importance of maintenance and decommissioning. As we have seen many times with traditional CDS, we must be transparent about CDS failures and disseminate the knowledge with the goal of improving the safety and trustworthiness of CDS globally [ 13 ]. As CDS experts, our vigilance will be even more important in the era of novel artificial intelligence-based CDS.

## References

[1] Schleyer T, Silva GS, Tirumalasetty J, Vedak S, Rajeevan H Muinul Islam M *et al.* Advancing Healthcare Sustainability through Decision Support Yearb Med Inform. 2025. https://doi.org/10.1055/s-0046-1816619 10.1055/s-0046-1816619 PMC13730714 42976801

[2] Lehmann CU, Subbiani V. Advances in Clinical Decision Support Systems: Contributions from the 2023 Literature. Yearb Med Inform. 2024 Aug;33(1):175-177.https://doi.org/10.1055/s-0044-1800739. 10.1055/s-0044-1800739 PMC12020640 40199303

[3] Lamy JB, Séroussi B, Griffon N, Kerdelhué G, Jaulent MC, Bouaud J. Toward a formalization of the process to select IMIA Yearbook best papers. Methods Inf Med. 2015;54(2):135-44.https://doi.org/10.3414/ME14-01-0031. 10.3414/ME14-01-0031 25396220

[4] Newsom KJ, Hall B, Martinez KA, Nelson S, Starostik P, Nguyen K. Integration of pharmacogenetic data in epic genomic module drives clinical decision support alerts. Front Pharmacol. 2024 Dec 20;15:1458095.https://doi.org/10.3389/fphar.2024.1458095. 10.3389/fphar.2024.1458095 PMC11695119 39759455

[5] Collins J, Karim M, Akcay B, Palaniappa N, Wong J. Implementing a Clinical Decision Support Tool to Reduce Operating Room Anesthetic Fresh Gas Flow: A Resident-Led, Sustainability-Focused Quality Improvement Initiative. J Grad Med Educ. 2024 Dec;16(6 Suppl):140-144.https://doi.org/10.4300/JGME-D-24-00074.1. 10.4300/JGME-D-24-00074.1 PMC11644587 39677900

[6] Kadura S, Eisner L, Lopa SH, Poulakis A, Mesmer H, Willnow N, Pigeon WR. Nudging towards Sleep-Friendly Health Care: A Multifaceted Approach on Reducing Unnecessary Overnight Interventions. Appl Clin Inform. 2024 Oct;15(5):1025-1039.https://doi.org/10.1055/a-2404-2344. 10.1055/a-2404-2344 PMC11602248 39191425

[7] Liu S, McCoy AB, Wright AP, Nelson SD, Huang SS, Ahmad HB, Carro SE, Franklin J, Brogan J, Wright A. Why do users override alerts? Utilizing large language model to summarize comments and optimize clinical decision support. J Am Med Inform Assoc. 2024 May 20;31(6):1388-1396.https://doi.org/10.1093/jamia/ocae041. 10.1093/jamia/ocae041 PMC11105133 38452289

[8] Rowland AF, Nguyen TH, Cunha PP, Ezhuthachan I, Orenstein E, Kandaswamy S, Lee T. Implementing a clinical decision support tool to increase early peanut introduction guidance. J Allergy Clin Immunol. 2024 Oct;154(4):988-995.e5.https://doi.org/10.1016/j.jaci.2024.07.005. 10.1016/j.jaci.2024.07.005 38996876

[9] Mercado O, Ruan A, Oluwalade B, Devine M, Gibbs K, Carr L. Leveraging Novel Clinical Decision Support to Improve Preferred Language Documentation in a Neonatal Intensive Care Unit. Appl Clin Inform. 2024 May;15(3):629-636.https://doi.org/10.1055/a-2332-5843. 10.1055/a-2332-5843 PMC11290947 38788772

[10] Zachariah JP, Singh T, Collinson S, Rahman J, Acosta AA, Campbell JF *et al.* Pediatric High Blood Pressure Recognition Associated With Electronic Decision Support: A Cohort Analysis. Hypertension. 2024 Dec;81(12):2501-2509. doi:10.1161/HYPERTENSIONAHA.124.23532. 10.1161/HYPERTENSIONAHA.124.23532 PMC11578764 39411867

[11] Fallon A, Haralambides K, Mazzillo J, Gleber C. Addressing Alert Fatigue by Replacing a Burdensome Interruptive Alert with Passive Clinical Decision Support. Appl Clin Inform. 2024 Jan;15(1):101-110.https://doi.org/10.1055/a-2226-8144. 10.1055/a-2226-8144 PMC10830237 38086417

[12] Solomonides AE, Koski E, Atabaki SM, Weinberg S, McGreevey JD, Kannry JL *et al.* Defining AMIA's artificial intelligence principles. J Am Med Inform Assoc. 2022 Mar 15;29(4):585-591. https://doi.org/10.1093/jamia/ocac006. 10.1093/jamia/ocac006 PMC8922174 35190824

[13] Applied Clinical Informatics. ACI Special Topics: CDS Failures. 2025 [cited 2025 30 June]https://www.thieme-connect.com/products/ejournals/journal/10.1055/s-00035026

